# Factors Influencing Practice Decisions Among Plastic Surgery Residents and Early-Career Plastic Surgeons in Canada

**DOI:** 10.1177/22925503241305637

**Published:** 2024-12-19

**Authors:** Sarah C. Hunt, Aaron Grant, Andrew M. Simpson

**Affiliations:** Division of Plastic and Reconstructive Surgery, 6221 Western University, London, ON, Canada

**Keywords:** academic surgery, community surgery, aesthetic surgery, career choice, residency training, plastic surgery workforce, chirurgien en milieu universitaire, chirurgie en milieu communautaire, chirurgie esthétique, choix de carrière, effectifs en chirurgie plastique

## Abstract

**Introduction:** Trends within the Canadian plastic surgery workforce demonstrated that most work in academic or medium-large community practice. Recent studies observed more plastic surgeons are incorporating aesthetics into their practice. This study aims to identify factors influencing how plastic surgery residents and early-career plastic surgeons in Canada choose their eventual practice with respect to practice type and practice location. **Methods:** A REDCap survey was distributed to plastic surgery residents and early-career plastic surgeons across Canada between February and May 2024. Demographics, training information, career information, and Likert-scale questions for factors involved in decision-making were surveyed. Data analysis included descriptive statistics and Fisher exact tests. **Results:** There were 45 residents and 30 early-career plastic surgeon respondents. Mixed practices that included aesthetics were the most popular practice types among residents (73%) and early-career surgeons (77%). Half (53%) of early-career surgeons were working in urban settings with more than 1 million people, and 44% of residents desired these locations. Hometown factors heavily influenced practice type and location (*P* < .0001), more than training experiences. Positive interactions (94%), operating time (90%), partner opinion (87%), and hospital resources (86%) were ranked as the most important factors involved in practice decisions. **Conclusion:** Mixed practices that include aesthetics were the most popular among our up-and-coming Canadian workforce, especially in urban settings. These changing practice trends may impact our ability as a specialty to adequately meet the needs of the Canadian population. Recruitment efforts should focus on promoting a supportive workplace and local environment, with adequate operating time and resources.

## Résumé

**Introduction :** Les tendances au sein des effectifs canadiens en chirurgie cardiaque ont démontré que la plupart travaillent dans des milieux universitaires ou dans des milieux communautaires de moyenne ou grande dimension. Selon de récentes études, plus de plasticiens intègrent l’esthétique à leur pratique. La présente étude visait à déterminer les facteurs qui influent sur les choix de pratique éventuelle des résidents en chirurgie plastique et des plasticiens en début de carrière au Canada pour ce qui est du type et du lieu de pratique. **Méthodologie :** Les résidents en chirurgie plastique et les plasticiens en début de carrière du Canada ont reçu un sondage REDCap entre février et mai 2024. Les chercheurs ont obtenu les renseignements démographiques, l’information sur la formation et la carrière, ainsi que des questions sur l’échelle de Likert pour sonder les facteurs qui contribuent aux prises de décision. L’analyse des données incluait les statistiques descriptives et la méthode exacte de Fisher. **Résultats :** Au total, 45 résidents en chirurgie plastique et 30 plasticiens en début de carrière ont répondu au sondage. Les pratiques mixtes qui incluaient l’esthétique étaient les plus populaires chez les résidents (73%) et les chirurgiens en début de carrière (77%). La moitié des chirurgiens en début de carrière (53%) travaillait dans des milieux urbains de plus d’un million d’habitants, et 44% des résidents souhaitaient travailler dans ces mêmes lieux. Des facteurs liés à la ville natale influaient fortement sur le type et le lieu de pratique (*P* < 0,0001), plus que les expériences de formation. Des interactions positives (94%), le temps d’opération (90%), l’opinion du partenaire (87%) et les ressources hospitalières (86%) étaient classés comme les facteurs les plus importants pour choisir sa pratique. **Conclusion :** Les pratiques mixtes qui incluent l’esthétique étaient les plus populaires chez les nouveaux effectifs canadiens, particulièrement en milieu urbain. Ces tendances peuvent influer sur la capacité à répondre de manière appropriée aux besoins de la population canadienne. Les efforts de recrutement devraient être orientés vers la promotion d’un milieu de travail et d’un environnement local positifs, qui offrent du temps opératoire et des ressources en quantité suffisante.

## Introduction

In recent decades, the workforce trends among plastic surgeons in Canada have been changing. Although the Canadian Society of Plastic Surgeons (CSPS) estimates there are enough plastic surgeons to meet the needs of the Canadian population,^1^ shortcomings of this target include lack of information on the geographic distribution of plastic surgeons across Canada,^1^ and lack of consideration for plastic surgeons working within the aesthetic realm.^1^

A survey distributed to CSPS members in 2017 demonstrated most plastic surgeons practice in urban settings, with half practicing in communities with over 1 million people.^1^ This may reflect trends whereby plastic surgeons who train in metropolitan areas establish practice in similar areas.^2^ A decrease in plastic surgeons working rurally has also been observed, with an estimated 2% of plastic surgeons working in rural Canada as of 2017,^1^ compared to 12% in 2007.^3^ Furthermore, an increasing number of plastic surgeons are incorporating aesthetics into their practice.^4,5^

Presently, there is a paucity of information describing how plastic surgeons choose their practice, as well as the factors underlying these decisions. There have been calls for these types of studies within the plastic surgery literature,^1,2^ but, to our knowledge, they have not yet been completed.

The specific objectives of this study were to determine practice intentions, trends, and the factors influencing these decisions among plastic surgery residents and early-career plastic surgeons (first 10 years of practice) in Canada, with respect to the type of practice (community vs academic vs aesthetic) and geographic location of practice.

## Methods

This prospective cohort study was approved by the institutional Health Science Research Ethics Board (#123864). Anonymized RedCAP surveys were distributed to all Postgraduate Year (PGY) 1-5 residents belonging to Canadian plastic surgery programs, and to early-career plastic surgeons in their first 10 years of practice who are members of CSPS.

Surveys included questions regarding demographic information, training information, career aspirations (residents) or current practice information (early-career plastic surgeons), and Likert scale-style questions relating to several factors that influence practice decisions. These factors were chosen based on those described in the literature among other surgical and nonsurgical specialties.^6–17^ Survey development involved completion of item generation, item reduction, formatting, and pretesting.^18,19^

Surveys were distributed over a 2-month period, with one reminder email sent after one month. Survey collection occurred between February-April 2024 (residents) and March-May 2024 (early-career plastic surgeons). Consent forms were completed prior to survey completion. At the end of the survey, participants had the opportunity to enter a prize draw to win Amazon gift cards.

Survey responses were organized in Microsoft Excel.^20^ Statistics, including descriptive statistics and Fisher Exact tests, were carried out using GraphPad Prism.^21^

## Results

### Survey Responses

Forty-five out of 151 plastic surgery residents in Canada completed the survey, representing a response rate of 30% (Table 1). There were 30 out of 106 early-career plastic surgeons in Canada who completed the survey, representing a response rate of 28%.

**Table 1.** Response Rates and Participant Demographics.

	*N* (%)
Response rates	
Residents	45 (29.8)
Early-career plastic surgeons	30 (28.3)
Stage of career	
Resident	45 (60.0)
Early-career plastic surgeon	30 (40.0)
Gender	
Male	39 (52.0)
Female	34 (45.3)
Nonbinary	1 (1.3)
Prefer not to say	1 (1.3)
Age	
25-29	20 (32.8)
30-34	19 (31.1)
35-39	15 (24.6)
≥40	7 (11.5)

### Demographics

There were more resident responses (60%) than early-career plastic surgeon responses (40%) (Table 1). There was slightly more male participation (52%) than female participation (45%). One participant identified as nonbinary. Most participants (88%) were under the age of 40.

### Future Training and Practice Intentions of Residents

Most residents (73%) intended to pursue mixed practices that include aesthetic surgery (Table 2). Mixed academic and aesthetic practice was the most popular practice type (42%), followed by mixed community and aesthetic practice (31%). Exclusive practice types were less popular among residents, with 16% interested in exclusively academic practice, 9% interested in exclusively aesthetic practice, and 2% interested in exclusively community practice. Academic practice was more popular than community practice (58% vs 33%). Residents preferred group practices (96%).

**Table 2.** Future Training and Practice Intentions of Residents.

	*N* (%)
Interest in fellowship	
Yes	36 (80.0)
No	2 (4.4)
Unsure	7 (15.6)
Fellowships of interest	
Microsurgery	24 (55.8)
Hand	21 (48.8)
Aesthetic	13 (30.2)
Craniofacial	12 (27.9)
Breast	12 (27.9)
Oncologic reconstruction	10 (23.3)
Peripheral nerve	9 (20.9)
Burn	6 (14.0)
Pediatrics	2 (4.7)
Gender affirming	1 (2.3)
Ideal future practice	
Exclusively aesthetic	4 (8.9)
Exclusively academic	7 (15.6)
Exclusively community	1 (2.2)
Mixed academic and aesthetic	19 (42.2)
Mixed community and aesthetic	14 (31.1)
Preference for group or solo practice	
Group	43 (95.6)
Solo	2 (4.4)
Population of ideal future practice location	
<50 000	0 (0.0)
50 000-99 999	3 (6.7)
100 000-249 999	5 (11.1)
250 000-499 999	9 (20.0)
500 000-999 999	8 (17.8)
≥1,000,000	20 (44.4)
Ideal future practice location	
Newfoundland	3 (6.7)
Prince Edward Island	3 (6.7)
Nova Scotia	5 (11.1)
New Brunswick	6 (13.3)
Quebec	10 (22.2)
Ontario	22 (48.9)
Manitoba	3 (6.7)
Saskatchewan	1 (2.2)
Alberta	11 (24.4)
British Columbia	17 (37.8)
Outside of Canada	16 (35.6)

Most residents (80%) were interested in pursuing a fellowship (Table 2). Fellowship options with the most interest included microsurgery (56%), hand (49%), aesthetics (30%), craniofacial (28%), and breast (28%). Fewer residents were interested in oncologic reconstruction (23%), peripheral nerve (20%), burns (14%), pediatrics (5%), and gender-affirming (2%). Residents wishing to pursue academic practice were more likely to want to pursue a fellowship (*P = .0352).

Nearly half (44%) of residents wanted to work in large cities with populations greater than 1 million (Table 2). Interest in practice locations decreased with increasing rurality. Those pursuing academic practice preferred to practice in larger city centres with populations greater than 500 000, compared to those pursuing community practice who preferred to work in smaller city centres with populations less than 500 000 (****P < .0001). Ontario was the most popular future practice destination (49%), followed by British Columbia (38%), and outside of Canada (36%).

### Current Practice Characteristics of Early-Career Plastic Surgeons

Mixed practices that include aesthetics were the most common practice types among early-career plastic surgeons (77%) (Table 3). More than half (57%) had a mixed community and aesthetic practice. Only 20% had mixed academic and aesthetic practice. Exclusive practice types were less common, with 13% having an exclusively academic practice, 7% having an exclusively community practice, and 3% having an exclusively aesthetic practice. Among this cohort, community practice was more common than academic practice (64% vs 33%).

**Table 3.** Current Practice Characteristics of Early-Career Plastic Surgeons.

	*N* (%)
Current practice	
Exclusively aesthetic	1 (3.3)
Exclusively academic	4 (13.3)
Exclusively community	2 (6.7)
Mixed academic and aesthetic	6 (20.0)
Mixed community and aesthetic	17 (56.7)
Group or solo practice	
Group	21 (67.7)
Solo	10 (32.3)
Communities since completion of training	
1	21 (70.0)
2	6 (20.0)
3 or more	3 (10.0)
Population of current community	
<50 000	2 (6.7)
50 000-99 999	3 (10.0)
100 000-249 999	3 (10.0)
250 000-499 999	6 (20.0)
500 000-999 999	0 (0.0)
≥1,000,000	16 (53.3)
Location of current community	
Newfoundland	0 (0.0)
Prince Edward Island	0 (0.0)
Nova Scotia	1 (3.3)
New Brunswick	0 (0.0)
Quebec	3 (10.0)
Ontario	12 (40.0)
Manitoba	1 (3.3)
Saskatchewan	1 (3.3)
Alberta	5 (16.7)
British Columbia	7 (23.3)
Territories	0 (0.0)
Outside of Canada	0 (0.0)
Population of previous community	
Smaller than current	5 (55.6)
Same size	4 (44.4)
Larger than current	1 (11.1)
Location of previous community	
Same province	8 (88.9)
Different province	2 (22.2)
Practice focus	
Aesthetic	19 (63.3)
Breast	17 (56.7)
Hand	17 (56.7)
General/Comprehensive	15 (50.0)
Microsurgery	10 (33.3)
Peripheral nerve	9 (30.0)
Oncologic reconstruction	8 (26.7)
Gender affirming	8 (26.7)
Pediatrics	6 (20.0)
Craniofacial	5 (16.7)
Burn	4 (13.3)
Other	3 (10.0)

The majority of respondents (68%) belonged to a group practice (Table 3). The most reported practice focuses included aesthetics (63%), breast (57%), hand (57%), and general/comprehensive plastic surgery (50%). Fewer early-career plastic surgeons had focuses in microsurgery (33%), peripheral nerve (30%), oncologic reconstruction (27%), gender affirming (27%), pediatrics (20%), craniofacial (17%), and burns (13%).

Over half of respondents (53%) worked in large cities with populations greater than 1 million (Table 3). There were fewer plastic surgeons working in more rural locations. Most early-career plastic surgeon respondents worked in Ontario (40%), British Columbia (23%), and Alberta (17%). Most settled in one community after training completion (70%). For those who changed practice locations early in their career, most remained in the same province (89%), and most moved to a community that was the same size (44%) or larger (56%) than their previous community.

### Influence of Living and Training Locations in Relation to Practice Decisions

The hometown population had the greatest influence on practice type. Those who chose community practice tended to come from smaller communities with less than 500 000 people, while those who chose academic practice tended to come from larger communities with greater than 500 000 people (***P = .0003). Training location populations for medical school, residency, and fellowship did not appear to influence chosen practice type. For those who completed community plastic surgery rotations during residency, the size of the community did not influence chosen practice type.

Most participants preferred to work in familiar locations. Residents desired to work in the same location as their hometown (80%), medical school (78%), or residency locations (80%). Only one-third of residents would consider moving elsewhere in Canada (33%) or internationally (36%) where they haven't previously lived or trained. Similarly, early-career plastic surgeons often set up practice in the same province as their hometown (67%), medical school (67%), residency (57%), or fellowship (42%).

### Influence of Educational Experiences in Relation to Practice Decisions

Among residents, year of training was not related to wanting a particular practice type (*P* = .7513). Of the 72% of respondents that completed a community plastic surgery rotation in residency, this exposure did not result in greater interest in community practice (*P* = .6069). Similarly, there was no association between completing an aesthetic surgery block and interest in an aesthetic practice (*P* > .9999). Among early-career plastic surgeons, 87% had completed a fellowship, which was not related to a particular practice type (*P* = .2680). Those choosing academic practice were more likely to have obtained a graduate degree (**P = .0094).

### Factors Influencing Practice Decisions

The most important factors influencing practice decisions agreed upon by residents and early-career plastic surgeons included positive interactions with colleagues (94%), operating time (90%), spouse or partner opinion (87%), and availability of hospital resources (86%) (Table 4). Other important factors agreed upon by three-quarters of respondents included community compatibility in relation to hobbies and interests (76%), employment and educational opportunities for spouses or partners (74%), proximity to family and friends (74%), quality of hospital facilities (74%), call requirements (73%), and working hours (73%). The least important factors included a narrow scope of practice (10%), COVID-19 pandemic experiences (4%), and a preference for working in a solo practice (3%).

**Table 4.** Factors Influencing Practice Decisions Among Residents and Early-Career Plastic Surgeons.

Factor	Total *N* (%)	Academic *N* (%)	Community *N* (%)	*P*-value
Positive interactions with colleagues	66 (94.3)	33 (91.7)	33 (97.1)	.6145
Operating time	63 (90.0)	32 (88.9)	31 (91.2)	>.9999
Spouse/partner's opinion	61 (87.1)	31 (86.1)	30 (88.2)	>.9999
Availability of hospital resources	60 (85.7)	32 (88.9)	28 (82.4)	.5079
Community compatible with hobbies/interests	53 (75.7)	27 (75.0)	26 (76.5)	>.9999
Community offers employment and educational opportunities for spouse/partner	52 (74.3)	25 (69.4)	27 (79.4)	.4171
Proximity to family and/or friends	52 (74.3)	27 (75.0)	25 (73.5)	>.9999
Quality of hospital facilities	52 (74.3)	32 (88.9)	20 (58.8)	.0058**
Call requirements	51 (72.9)	25 (69.4)	26 (76.5)	.5955
Working hours	51 (72.9)	25 (69.4)	26 (76.5)	.5955
Income potential	48 (68.6)	27 (75.0)	21 (61.8)	.3050
Preference for working in a group practice	46 (65.7)	27 (75.0)	19 (55.9)	.1311
Broad scope of practice	42 (60.0)	21 (58.3)	21 (61.8)	.8110
Community offers good schools for children	39 (56.5)	22 (61.1)	17 (51.5)	.4724
Complexity of cases	37 (52.9)	27 (75.0)	10 (29.4)	.0003***
Career advancement opportunities	36 (51.4)	26 (72.2)	10 (29.4)	.0007***
Teaching opportunities	36 (51.4)	26 (72.2)	10 (29.4)	.0007***
Previous experiences living and/or working in community	33 (47.8)	17 (48.6)	16 (47.1)	>.9999
Availability of office space	33 (47.1)	17 (47.2)	16 (47.1)	>.9999
Patient population	23 (32.9)	14 (38.9)	9 (26.5)	.3154
Recruitment incentives	23 (32.9)	12 (33.3)	11 (32.4)	>.9999
Familiarity with healthcare system	17 (24.3)	7 (19.4)	10 (29.4)	.4080
Research opportunities	15 (21.4)	14 (38.9)	1 (6.7)	.0003***
Familiarity with hospital	11 (15.7)	5 (13.9)	6 (17.7)	.7495
Working with an underserved population	9 (12.9)	7 (19.4)	2 (5.9)	.1522
Narrow scope of practice	7 (10.0)	5 (13.9)	2 (5.9)	.4297
COVID-19 pandemic experiences (both home- and work-related)	3 (4.3)	1 (2.8)	2 (5.9)	.6087
Preference for working in a solo practice	2 (2.9)	2 (5.6)	0 (0.0)	.4936

*N* = number of participants ranking factor as “important” or “very important” (Likert 5-6).

** *P* < .01.

*** *P* < .001.

Respondents who preferred academic practice types were more likely to place a higher degree of importance on the quality of hospital facilities (**P = .0058), complex cases (***P = .0003), career advancement opportunities (***P = .0007), teaching opportunities (***P = .0007), and research opportunities (***P = .0003) (Table 4).

## Discussion

Most residents and early-career plastic surgeons in Canada are interested in mixed practices that include aesthetics, pointing to an increasing interest in aesthetics than previously observed. In a 2017 survey of Canadian plastic surgeons, only 26.6% reported mixed practices and 24% reported varying degrees of aesthetic practice.^1^ Our study determined that 73% of residents desire mixed practices while 77% of early-career plastic surgeons are currently in mixed practices. A survey in the United States (US) found that 92% of residents intended to practice aesthetic surgery upon training completion, confirming that this trend is observed on a more global scale.^22^ Others have observed it is becoming increasingly common to see early-career plastic surgeons transition to predominant or full-time aesthetic practice, a phenomenon coined the “cosmetic vacuum”.^4^ It has been suggested that this transition may interfere with our ability as plastic surgeons to adequately serve the public.^4^ Although it was previously suggested that Canada has enough plastic surgeons to meet the ideal surgeon-to-population ratio set by the CSPS,^1^ this target does not account for these mixed practice models and should therefore be adjusted to account for this trend.

This study found that 58% of residents are interested in academic practice, which is consistent with a US-based study which found that 62% of residents are interested in academic practice.^23^ Our cohort placed a higher degree of importance on complex cases, career advancement opportunities, teaching and research opportunities, and the quality of hospital facilities. Similar observations have been made among US plastic surgery residents interested in academic careers, who listed educational roles and complexity as attractive features of academic practice.^23^

Regarding practice location, this study observed that urban practices continue to be the most popular, and our workforce is projected to continue the trend observed in 2017, which found that 47% of plastic surgeons worked in urban centres with populations greater than 1 million.^1^ Our study determined that urban practice was associated with academic practice, which is expected given that academic centres tend to exist in large urban areas. It was also observed that more than half of early-career plastic surgeons who changed their practice location in the first 10 years tended to move their practices from smaller to larger communities. These findings raise concern regarding adequate plastic surgeon coverage of smaller communities, which has previously been flagged as a potential workforce issue in Canada.^1^

Residents and early-career plastic surgeons tended to choose practice locations with elements of familiarity. A striking finding of this study was that the population size of one's hometown was the best predictor of desired practice location, whereby those who came from larger communities tended to prefer academic practice in larger communities, while those who came from smaller communities tended to prefer community practice in smaller communities. Respondents also preferred to stay within a familiar province, with more than half preferring to stay in the same province as their hometown, medical school, or residency location. This finding contrasts a US-based study which found that most plastic surgeons established practices far from their training institutions.^2^

A previous study showed that most plastic surgeons working rurally have experience in rural settings prior to practice.^24^ Our study demonstrates that growing up in these settings is the best predictor for practicing in these settings. This study also demonstrated that interest in practice locations dwindled with increasing rurality, raising concern for coverage of these regions from a workforce perspective. Although previous studies have called for rural community plastic surgery rotations to promote practice in these areas,^24^ our study suggests that these rotations are not effective in promoting community practice in smaller communities. Based on the findings of this study, recruiting plastic surgery trainees from smaller communities may be the best option for filling these gaps in our workforce.

Finally, respondents in this study agreed that the most important factors influencing practice decisions, regardless of practice type, involved having a supportive local network and necessary resources to support their work. Positive interactions with colleagues were the most important factor, suggesting that a welcoming, collegial, and supportive work environment is paramount in recruiting plastic surgeons. Hospitals must also ensure they are offering new recruits adequate operating time and supporting plastic surgeons with the resources they need to carry out their jobs. Finally, having a supportive network outside of work, including spouse or partner support, family and friends close by, or local hobbies, are all important factors in recruiting plastic surgeons. These were more important than other common aspects of contract negotiation such as income, recruitment incentives, call requirements, and working hours. Those involved with hiring and retention of plastic surgeons may consider these factors when advising and evaluating potential candidates.

### Limitations

The results of this study were mostly limited by sample size and response rate. Low response rates from our population of interest may impact the validity of our study results and limit our ability to draw accurate conclusions. Furthermore, most respondents were from Ontario, which may fail to incorporate diverse perspectives from across Canada. This study also examined a limited number of factors influencing practice and is unlikely to adequately capture all nuanced factors involved in practice decisions. Finally, the results of this study capture the views of a Canadian cohort and may not be generalizable to other populations of plastic surgeons elsewhere in the world.

## Conclusions

Mixed practice styles that incorporate aesthetics are increasingly popular among plastic surgery residents and early-career plastic surgeons in Canada, highlighting a trend that may impact our ability as a specialty to meet the needs of the Canadian population. The urban practice continues to be popular and raises concerns regarding the adequate geographic distribution of plastic surgeons in our workforce. Plastic surgery trainees and early-career plastic surgeons in Canada emphasize social supports, community compatibility, hospital resources and operating time as important factors implicated in practice decisions and should therefore be a major focus of recruitment.
